# Elevated plasma levels of TNF-alpha and Interleukin-6 in patients with diastolic dysfunction and glucose metabolism disorders

**DOI:** 10.1186/1475-2840-8-58

**Published:** 2009-11-12

**Authors:** Wilfried Dinh, Reiner Füth, Werner Nickl, Thomas Krahn, Peter Ellinghaus, Thomas Scheffold, Lars Bansemir, Alexander Bufe, Michael Coll Barroso, Mark Lankisch

**Affiliations:** 1 Helios Clinics Wuppertal, Heart Center, Germany; 2 Institute for Heart and Circulation Research, University Witten/Herdecke, Germany; 3 Target Discovery, Bayer Schering Pharma, Wuppertal, Germany; 4 CoroVital, Institute of Sports Cardiology and Science, Wuppertal, Germany

## Abstract

**Background:**

Diabetes mellitus (DM) has reached epidemic proportions and is an important risk factor for heart failure (HF). Left ventricular diastolic dysfunction (LVDD) is recognized as the earliest manifestation of DM-induced LV dysfunction, but its pathophysiology remains incompletely understood. We sought to evaluate the relationship between proinflammatory cytokine levels (TNF-alpha, IL-6) and tissue Doppler derived indices of LVDD in patients with stable coronary artery disease.

**Methods:**

We enrolled 41 consecutive patients (mean age 65+/-10 years) submitted for coronary angiography. Echocardiographic assessment was performed in all patients. Pulsed tissue Doppler imaging was performed at the mitral annulus and was characterized by the diastolic early relaxation velocity Em. Conventional transmitral flow was measured with pw-doppler. Early (E) transmitral flow velocity was measured. LVDD was defined as E/Em ratio ≥ 15, E/Em 8-14 was classified as borderline. Plasma levels of TNF-alpha and IL-6 were determined in all patients. A standardized oral glucose tolerance test was performed in subjects without diabetes.

**Results:**

Patients with E/Em ratio ≥ 15, classified as LVDD and those with E/Em ratio 8-14 (classified as borderline) had significantly higher IL-6 (P = 0,001), TNF-alpha (P < 0,001) and NT-pro- BNP (P = 0,001) plasma levels compared to those with normal diastolic function. TNF-alpha and IL-6 levels remains significantly elevated after adjustment for sex, age, left ventricular ejection function, body mass index, coronary heart disease, smoking, hypertension and diabetes mellitus with linear regression analysis. Furthermore, in subjects LVDD or borderline LV diastolic function, 75% had diabetes or IGT, respectively. When subjects without diabetes were excluded, both IL-6 (P = 0,006) and TNF-alpha (P = 0,002) remained significantly elevated in subjects with E/Em ratio ≥ 15.

**Conclusion:**

This study reveals that increased plasma levels of IL-6 and TNF-alpha were associated with LVDD. These findings suggest a link between low-grade inflammation and the presence of LVDD. An active proinflammatory process may be of importance in the pathogenesis of diastolic dysfunction.

## Background

Diastolic heart failure and diabetes mellitus (DM) are contributors to mortality, hospitalization and medical costs in health care systems worldwide. Cardiovascular disease is the leading course of death among subjects with diabetes, accounting for over 60% of mortality. While DM is uniformly recognized as an important risk factor for the development of coronary artery disease and its complications, it is less well acknowledged that DM is a powerful and independent risk factor for the development and prognosis of heart failure independent of coronary artery disease (CAD) [1]. Left ventricular diastolic dysfunction (LVDD) is considered a precursor of systolic heart failure and diabetic cardiomyopathy [2,3] and is common in the community and especially in diabetic patients [4,5].

There is increasing evidence that inflammation is involved in the pathophysiology of heart failure and diabetes [6-9]. Most recent studies have linked insulin- resistance with TNF-α and IL6 and those studies have shown that a measure of proinflammation is predictive for type 2 diabetes. Furthermore, increased circulating concentrations of IL-6 and TNF-α were found in Type 2 DM and impaired glucose tolerance [10-12].

Little information is available about whether inflammation is involved in the development of LVDD as the earliest stage in the development of heart failure.

Advantages in echocardiographic techniques have made it possible to comprehensively access LV diastolic function [13]. Tissue Doppler imaging is a simple, reproducible and widely available noninvasive tool for the assessment of alterations of left ventricular (LV) diastolic function. A recent guideline of the American Society of Echocardiography (ASE) implemented TDI techniques as the basic principle to diagnose diastolic heart failure [14].

This study was designed to explore the possible association between LVDD and proinflammation reflected by IL-6 and TNF-α levels in subjects with stable coronary artery disease.

## Methods

The present study is a substudy of a larger survey in which we investigated the relationship between diastolic dysfunction and cardiovascular autonomic neuropathy in 150 patients submitted for coronary angiography.

41 subjects submitted for coronary angiography for stable or suspected coronary artery disease (CAD) were consecutively enrolled in this substudy. The protocol was approved by the local ethics committee, and signed informed consent was obtained from all patients. We excluded patients with hypertrophic obstructive cardiomyopathy, moderate-to-severe valvular disease, uncontrolled hypertension, atrial fibrillation or other severe arrhythmias. Oral glucose tolerance tests (OGTTs) were carried out according to the World Health Organization protocol as previously described. Echocardiography for the diagnosis of diastolic dysfunction was performed on a Philips IE33 Ultrasound System machine. Conventional transmitral flow was measured with pw-doppler. Early (E), atrial (A) transmitral peak flow velocities and the ratio (E/A) were measured. Pulsed wave TDI was performed at the junction of the LV wall with the medial mitral annulus end-expiratory. Early diastolic velocity (Em) was recorded. Ratio of E/Em was calculated. The E/Em ratio was used to classify LVDD. LVDD was defined as E/Em ratio ≥ 15, borderline diastolic function as E/Em between 8-14 and normal diastolic function as E/Em < 8 according to the guidelines of the ASE [14].

Interleukin-6 and TNF-α were measured with high-sensitivity enzyme linked immunoassay [14]. NT-pro BNP was measured by a commercial available Immuno-Assay (Roche Diagnostics^®^).

Autonomic nervous function was assessed according to the guidelines of German diabetes association. Spectral analysis with Fourier transformation was performed. Therefore a commercially computer program (VariaCardio TF4-System) was used. The following tests were performed: (1) coefficient of variation of R-R intervals at rest, (2) spectral power in the very-low-frequency band, (3) spectral power in the LF band, (4) HRV during deep breathing, (5) maximum/minimum 30:15 ratio, (6) valsalva ratio, (7) postural change in systolic blood pressure. CAN is defined as the presence of ≥ 3 abnormalities among these 7 parameters.

### Statistical analysis

All analyses were performed using SPSS statistical software (SPSS 17.0, Chicago, IL). The data are presented as mean +- SD unless otherwise specified. A *p *value ≤ 0.05 was considered statistically significant. Comparison among the 2 groups of subjects for various parameters was performed by 1-way analysis of variance (ANOVA or t-test) when appropriate. Pearson's linear correlation coefficients were calculated for pairs of continuous variables, Spearman's correlations coefficient was used when data were not normally distributed. We first analyzed associations without any adjustments and then with adjustments for potential confounders by multiple linear regression for continuous and logistic regression for categorical variables.

## Results

### Demographics and clinical variables

**Figure 1:**
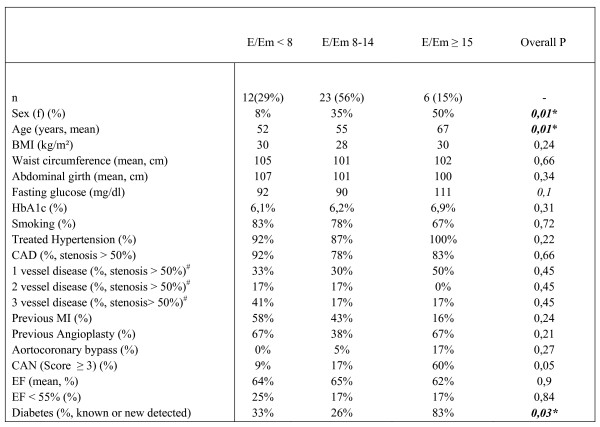
**Demographics and clinical variables**. Demographics and clinical variables plotted against E/Em ratio. CAN = cardiovascular autonomic neuropathy, BMI = body mass index, CAD = coronary artery disease, MI = myocardial infarction, DM = Diabetes mellitus. * = significant (P < 0,05). ^# ^= no history of aortocoronary bypass surgery.

The demographic variables of subjects with normal, borderline and increased E/Em ratio are shown in Figure 1. Subjects with E/Em > 15 were significantly older with a higher prevalence of DM. The prevalence of coronary heart disease, systolic dysfunction and hypertension as possible confounders contributing to diastolic dysfunction was similar between the groups.

### Glucose tolerance test

In subjects without DM, an oGTT was performed. 21 (29%) of patients enrolled in the study had a history of diabetes. The oGTT showed impaired fasting glucose (IGT) or new detected Diabetes (NDM) in 32 (63%) of the included patients, only 11 (27%) of subjects enrolled in the study had normal glucose metabolism (NGT). In subjects with E/Em ratio ≥ 15, no patient was found with NGT. 16,7% had IGT or NDM and 66,7% known diabetes, respectively. Fasting glucose was 111 mg/dl in subjects with E/Em ratio ≥ 15 vs. 92 mg/dl and 90 mg/dl in those with borderline or normal E/Em ratio (P = 0,1), whereas HbA1c was 6,9%, 6,2% and 6,1%, respectively (P = 0,3).

### Proinflammatory cytokines and LVDD

The mean Em velocity was 8,4 cm/s in subjects with E/Em ratio < 8, 7,0 cm/s in those with E/Em ratio 8-15 and 5,3 cm/s in those with E/Em ratio ≥ 15, respectively.

**Figure 2:**
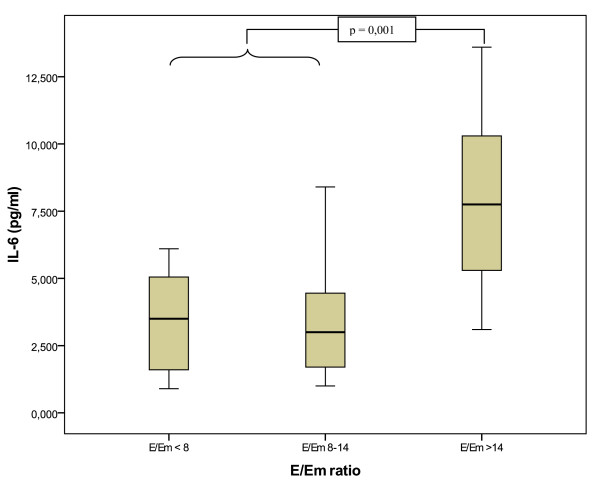
**E/Em ratio and IL-6 levels**. IL-6 levels are significantly elevated in subjects with LVDD. E = Early mitral inflow velocity, Em = early mitral tissue relaxation velocity.

**Figure 3:**
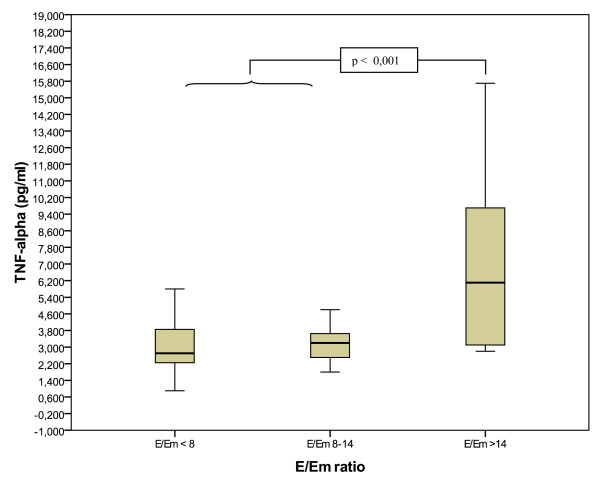
**E/Em ratio and TNF-α levels**. TNF-α levels are significantly elevated in subjects with LVDD. E = Early mitral inflow velocity, Em = early mitral tissue relaxation velocity.

E/Em ratio was positively correlated with TNF-α levels (r = 0,34, P = 0,05, Spearman-Test). In the post-hoc analysis, patients with E/Em ratio > 15, classified as LVDD, had significantly higher IL-6 (7,9 pg/ml vs. 3,4 pg/ml; P = 0,001), TNF-α (7,2 pg/ml vs. 3,1 pg/ml P < 0,001) compared to those with normal diastolic function (E/Em < 8; figure 2*and *3). There was no statistically significant difference between the borderline group and those with normal diastolic dysfunction (P > 0,5). TNF-α (B = 1,7, P = 0,01) and IL-6 (B = 1,51; P = 0,04) levels remains significantly elevated after adjustment for sex, age, left ventricular ejection function, body mass index, coronary heart disease, smoking, hypertension and diabetes mellitus with linear regression analysis. When subjects without diabetes were excluded, both IL-6 (P = 0,006) and TNF-alpha (P = 0,002) remained significantly elevated in subjects with E/Em ratio ≥ 15.

### Cardiovascular autonomic neuropathy (CAN)

New detected cardiovascular autonomic neuropathy (CAN+) was found in 22% of the study group. Although statistically not significant, 60% of subjects with diastolic dysfunction defined as E/Em ratio ≥ 15 were found CAN+, whereas only 9% of subjects with normal left ventricular diastolic function were CAN+. In the subgroup with borderline diastolic function, 20% were diagnosed as CAN+. Of note, in patients CAN+, IL6 levels were significantly higher than in those CAN- (6,1 pg/ml vs. 3,7 pg/ml, P = 0,42). Although statistically not significant, TNF-α levels were also numerical higher (4,4 pg/ml vs. 3,5 pg/ml, P = 0,42).

### NT-pro-BNP and LVDD

NT-pro-BNP levels were significantly higher in those with E/Em ratio > 15 compared to those with borderline E/Em ratio or normal diastolic function (1220 pg/ml vs. 192 pg/ml vs. 277 pg/ml, P = 0.001). In diabetics, NT-pro- BNP levels were 608 pg/ml vs. 229 pg/ml in non diabetics (P = 0,06).

## Discussion

We were able to demonstrate that LVDD and proinflammatory cytokines are associated in subjects with normal left ventricular systolic function. In particular increased plasma levels TNF-α and IL-6 seem to be correlated with impaired LVDD and more advanced left ventricular diastolic dysfunction. These data provide clinically important information on systemic immune abnormalities in subjects with LVDD. The Framingham Heart Study was the first to demonstrate an increased risk of heart failure in patients with diabetes [1]. Since then, additional studies, including SOLVD [15] and HOPE [16], have identified diabetes as a major risk factor for the development of heart failure. Conversely, the presence of heart failure was identified as a possible risk factor for diabetes [17]. Several studies have demonstrated evidence for preclinical left ventricular diastolic dysfunction (LVDD) as the first manifestation of myocardial involvement in diabetic patients [3]. Although myocardial changes can be detected with echocardiography, even before the onset of hemodynamic abnormalities in subjects with normal conventional echocardiography, LVDD is often not diagnosed in clinical practice. On the other hand, there is increasing evidence that inflammation is involved in the pathophysiology of heart failure and diabetes [6-9]. Inflammation has become one of the central themes in the pathogenesis of systolic heart disease over the past decade. So far there have been few data on participation of inflammatory factors in the development of diastolic dysfunction.

It has been demonstrated that IL-6 shows cardiodepressive properties [18]. In patients with systolic heart failure, IL-6 and TNF-α are associated with functional NYHA class [8]. Furthermore, IL-6 and TNF-α have been shown to be independent predictors of mortality in heart failure [19]. On the other hand, a recent study which followed patients with symptomatic systolic heart failure, E/Em ratio was associated with an increased risk of death or heart transplantation. A E/Em ratio > 17 had a mortality of approximately 40% vs. 5% in those with an E/Em ratio of less than 17 (p < 0,001) [20]. From at al. demonstrated that there is an association between duration of DM and LVDD and that an increasing E/Em ratio is associated with all-cause mortality in diabetic patients [21]. In this study, duration of DM ≥ 4 years was correlated with significant LV diastolic dysfunction and LVDD was predictive of all-cause mortality independent of hypertension and CAD. Recently Mogelvang et colleagues showed that in the general population, LVDD by TDI is a powerful and independent predictor of death, even in the subgroup with normal conventional echocardiography [22]. Focusing on the elderly subjects, who are prone for diabetes and its complications, diastolic dysfunction is an independent predictor of cardiovascular events [23]. Therefore, we speculate that the association of elevated proinflammatory cytokines with LVDD might put the patients at higher risk for the progression of symptomatic diabetic cardiomyopathy.

Proinflammatory cytokines are capable of modulating cardiovascular function by various mechanisms. It is now known that virtually every nucleated cell type in the myocardium, including the cardiac myocyte, is able to secrete proinflammatory cytokines in response to various myocardial damage or stressors. The expression of these cytokines can occur in absence of systemic immune activation.

They partly act in a negative inotropic manner and cause changes in turnover of the extracellular matrix resulting in myocardial fibrosis. The proinflammatory cytokine TNF-α induces cardiodepressive effects and causes apoptosis [24]. The development of progressive cardiomyocyte apoptosis plays a critical role on the left ventricular geometry and the adverse cardiac remodeling that occurs in the setting of sustained inflammation.

There are some limitations in the present study. The consensus paper of the ESC [25] deals with the term diastolic heart failure, referring to subjects with heart failure. In our study, diastolic dysfunction was defined in asymptomatic patients using TDI techniques. Nevertheless, TDI is the most sensitive and widely available echocardiographic tool for the assessment of LV diastolic function [14]. Furthermore, the fact that Em- velocity and E/Em are highly correlated with age might impair their predictive abilities in high risk population like ours in which age had an impact on the risk of cardiovascular and diabetes- related complications.

Moreover, we did not measure LA volume index and pulmonary vein velocities. These additional echo data might have been of interest especially in the cohort with E/Em ratio 8-14.

## Conclusion

Our study shows for the first time, to the best of our knowledge, a relationship between proinflammatory cytokines and abnormalities of diastolic performance in patients without clinical heart failure. The development of diastolic dysfunction and its possible sequelae diabetic cardiomyopathy is likely to be multifactorial, with putative mechanism including metabolic disturbances, insulin resistance, myocardial fibrosis, endothelial dysfunction, autonomic dysfunction and myocyte damage. Proinflammatory cytokines are involved in most if not all of these pathophysiological changes and might be a link between these abnormalities. At present, it is not clear which mechanism can explain the association between diastolic abnormalities and IL-6 and TNF-α-levels. The relationship between LV diastolic performance and the activation of immunoinflammatory system should be studied more thoroughly in the future both on experimental and clinical grounds.

## Competing interests

The authors declare that they have no competing interests.

## Authors' contributions

WD conceived of the study, participated in the study and drafted the manuscript and performed statistical analysis. RF participated in echocardiographic studies and participated in the study design of the study. LB participated in echocardiographic studies. AB participated in echocardiographic study. WN carried out the plasma analysis of IL6, TNF-α and BNP. TK carried out the plasma analysis of IL6, TNF-α and BNP. PE carried out the plasma analysis of IL6, TNF-α and biomarkers. TS participated in the study design of the study and performed statistical analysis. MCB participated in echocardiographic studies. ML participated in the study design and coordination and helped to draft the manuscript. All authors read and approved the final manuscript.
